# Minimally invasive treatment of peristomal metastases from gastric cancer at an ileostomy site by electrochemotherapy

**DOI:** 10.2478/raon-2013-0051

**Published:** 2013-10-08

**Authors:** Luca G. Campana, Marco Scarpa, Antonio Sommariva, Elena Bonandini, Sara Valpione, Leonardo Sartore, Carlo R. Rossi

**Affiliations:** 1 Sarcoma and Melanoma Unit, Veneto Institute of Oncology (IOV-IRCCS), Padova, Italy; 2 Surgical Oncology Unit, Veneto Institute of Oncology (IOV-IRCCS), Padova, Italy; 3 Department of Pathology, University of Padova, Padova, Italy; 4 Melanoma Cancer Unit, Veneto Institute of Oncology (IOV-IRCCS), Padova, Italy; 5 Plastic Surgery Unit, University of Padova, Padova, Italy

**Keywords:** stomach neoplasms, ileostomy, electrochemotherapy, skin care, palliative care

## Abstract

**Background:**

Peristomal metastases are rare, but potentially associated with relevant morbidity. Surgical resection, followed by stoma relocation, represent the gold standard in most patients. We describe electrochemotherapy (ECT), a minimally invasive method for locally-enhancing drug delivery by means of electric pulses, as an alternative approach.

**Patient and methods:**

A 49-year-old man with advanced gastric cancer developed skin metastases around an ileostomy site. The ulcerated and oozing tumor growth impaired patient’s quality of life due to continuous trouble in fitting the ostomy appliance, its poor adherence and consequent stools spillage. ECT consisted of a 20-minute course under mild general sedation. A bleomycin bolus of 15 000 IU/m^2^ was followed by the percutaneous application of multiple, 1.5 ms -long electric pulses by means of a needle electrode.

**Results:**

Post ECT course was uneventful and the patient was discharged on the same day. After one week, tumor nodules were flattened and partial tumor regression was appreciable at one-month follow-up. More importantly, peristomal skin conditions significantly improved, thus allowing for an effective application of the ostomy appliance during the following moths, until patient’s death.

**Conclusions:**

This report suggests the feasibility of ECT as a minimally invasive approach for peristomal tumors. In selected cases, ECT, by achieving a rapid tumor control, may ensure effective ostomy management and preserve patients’ quality of life.

## Introduction

**FIGURE 1.**
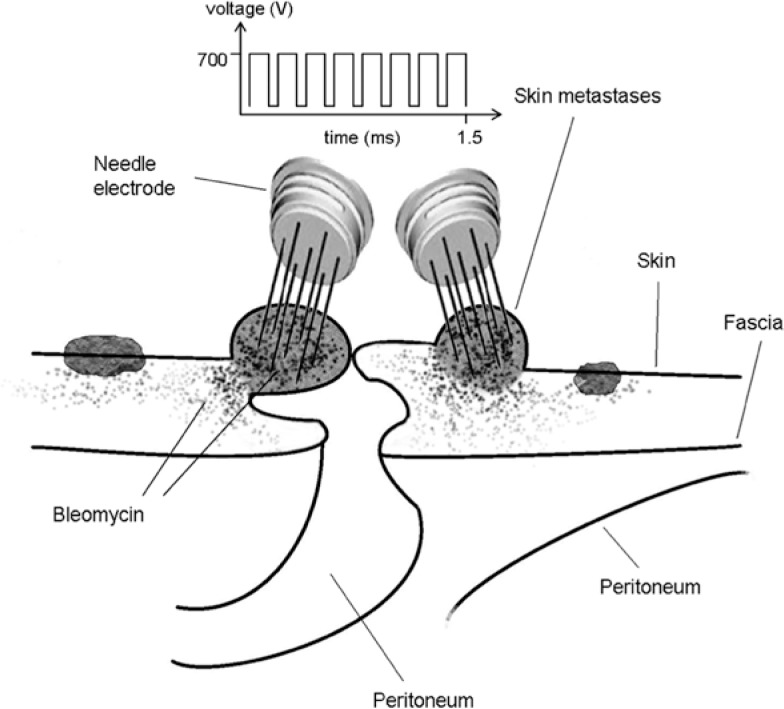
Electrochemotherapy of peristomal skin metastases. Eight minutes after intravenous injection, bleomycin molecules are equally distributed in body tissues. Tumor nodules are briefly exposed to a train of eight consecutive, high voltage (1000 V/cm), square-wave, 100-ms electric pulses, delivered at a repetition frequency of 5000 Hz by means of a needle electrode inserted into tumor tissue and connected to a pulse generator. As a consequence, transient pores open on the cell membrane and enable bleomycin concentration and entrapment, thus increasing its cytotoxic activity.

Despite the introduction of combined treatment strategies, gastric cancer (GC) remains one of the leading causes of cancer-related death worldwide.^1^ The most common metastatic sites are lymph nodes, liver, ovary and peritoneal cavity. The occurrence of skin metastases is a rare event, generally found at a very late stage of disease 2–4 and, occasionally, as the initial clinical manifestation.^5^–^8^ Isolated superficial metastases have been also described following invasive procedures (*i.e.* laparoscopic surgery).^9^,^10^ Peristomal metastases represent an even rarer, but challenging finding, due to possible bowel obstruction and trouble in ostomy management. Wide local excision and stoma relocation, when feasible, represent the gold standard treatment for these patients. Unfortunately, only few of them are still considered resectable when skin metastases occur, due to disease extension and poor general conditions. Electrochemotherapy (ECT) is a minimally-invasive approach which is gaining increasing acceptance in patients with unresectable or refractory superficial metastases, thanks to its sustained antitumor activity in different tumor histotypes, rapid patient’s recovery and favorable short-term outcomes.^11^,^12^ ECT mechanism relies on the association of an anticancer agent, bleomycin (BLM) or cisplatin (CDDP), with transient tumor permeabilization by means of brief, high-voltage, electric pulses (electroporation).^13^ The drug can be administered as an intravenous bolus or, alternatively, by the intratumoral route, according to disease burden.^14^ Tumor electroporation is achieved by the application of suitable plate or needle electrodes. Electric pulses open multiple, reversible pores on cell membrane, throughout which an increased number of chemotherapy molecules entry into the cell and exert their selective cytotoxic activity (Figure 1). We here report on the first case featuring ECT as an alternative treatment in a patient with symptomatic skin metastases from GC at an ileostomy site.

## Case report

**FIGURE 2.**
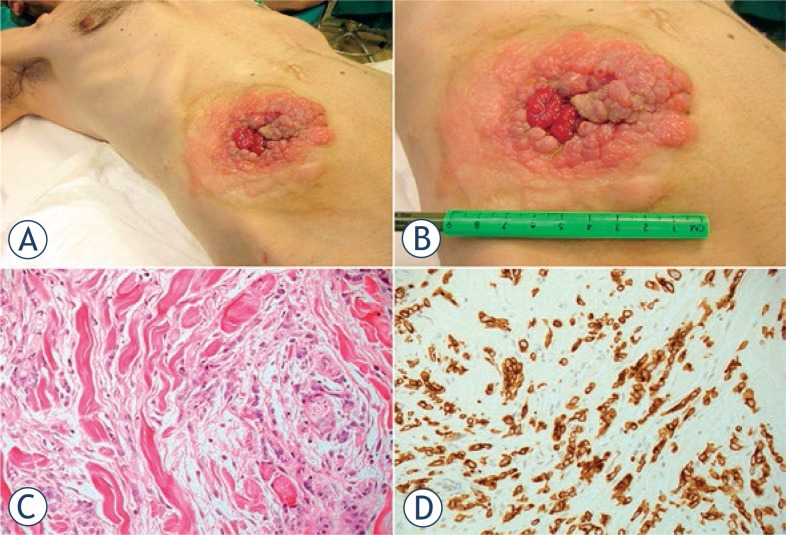
Peristomal skin tumor infiltration from gastric cancer at the ileostomy site. Baseline clinical presentation (**A, B**). The histological examination (E.E.) showed a dermal infiltration of neoplastic cells with atypical and eccentric nuclei, with nucleoli and pale cytoplasm and a signet ring aspect (**C**), immunoreaction for CAM5.2 (**D**).

**FIGURE 3.**
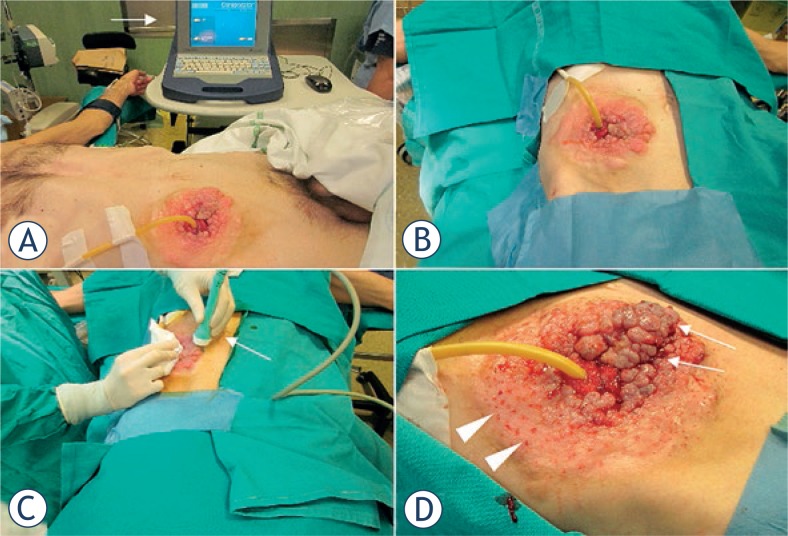
Electrochemotherapy treatment. The patient in the operating room and the electric pulse generator (*arrow*) (**A**). The ECT field (**B**). The application of electric pulses by means of the needle electrode (*arrow*) (**C**). Early postoperative skin conditions, with slight erythema at the electrode insertion sites (*arrowheads*) and partial tumor bluish coloration due to voltage-induced vasoconstriction (*arrows*) (**D**).

**FIGURE 4.**
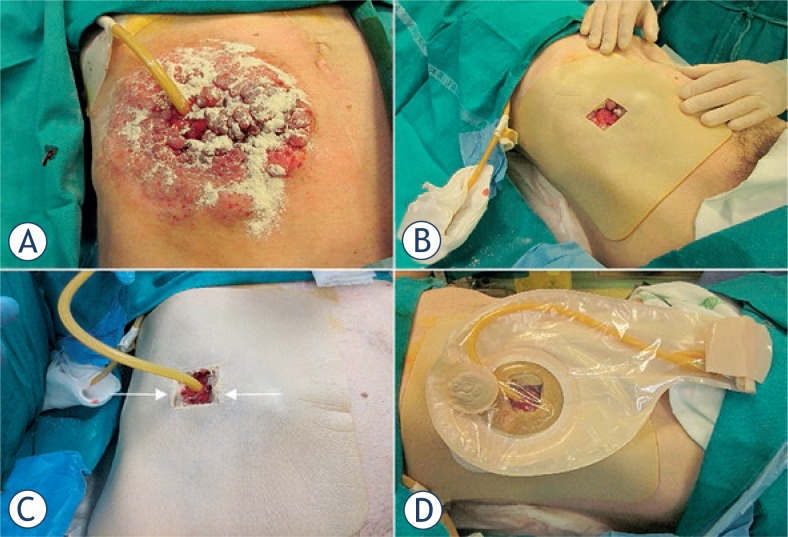
Post treatment wound dressing. Stoma powder application to absorb moisture from broken skin (**A**). Covering with an hydrocolloid dressing (**B**). Insertion of a silicon tube into the stoma to drain the stools and sealing of the hydrocolloid dressing with a stoma paste (*arrows*) (**C**). Effective application and sealing of the ileostomy flange and bag (**D**).

**FIGURE 5.**
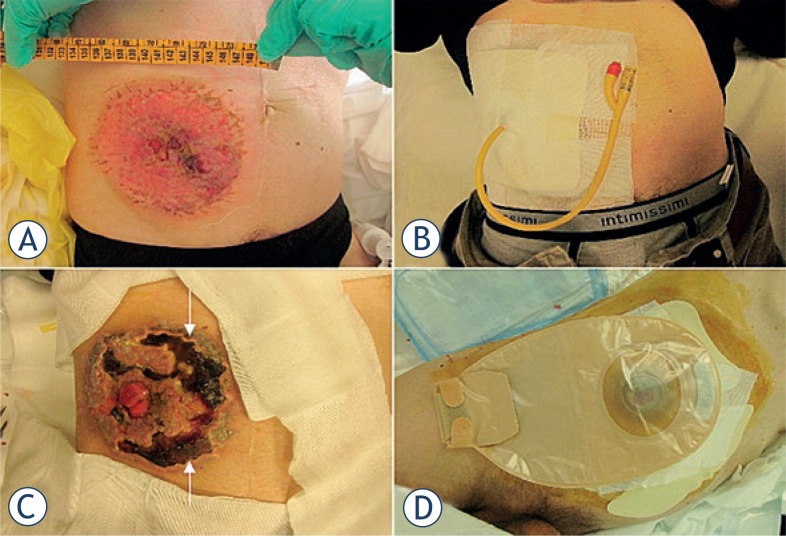
Follow-up. Clinical presentation one week after ECT (**A**). Application of the ileostomy bag on the hydrocolloid dressing (**B**). Clinical presentation three weeks after ECT (**C**). Effective application of the ileostomy bag on the abdominal wall (**D**).

A 49-year-old man with peritoneal carcinomatosis from GC, who previously required the construction of an end-ileostomy due to bowel obstruction, presented with multiple peristomal metastases that were confluent in a 10 × 10 cm area on the abdominal wall (Figure 2). In 2008 the patient was diagnosed with stage IV GC with peritoneal dissemination. He received 3 cycles of epirubicin, oxaliplatin and 5-fluorouracil, before undergoing total gastrectomy, D2 lymphadenectomy, distal pancreatectomy and splenectomy. Histopathological examination was T4aN3aM1, signet ring cells, HER2 negative, gastric adenocarcinoma. Additional 5 cycles of adjuvant chemotherapy were administered after surgical treatment. In April 2010, chemotherapy with epirubicin, oxaliplatin and 5-fluorouracil was started and then shifted to docetaxel, cisplatin plus 5-fluorouracil. In September 2011, an ileostomy was created in the lower right abdominal quadrant in order to palliate the progressively worsening symptoms of bowel obstruction. Third line monochemotherapy with irinotecan was ongoing at the time of our first evaluation, on October 2011. Given patient’s low performance status according to the Eastern Cooperative Oncology Group scale, peritonectomy associated with intraperitoneal chemotherapy was deemed contraindicated. On the other hand, peristomal tumor growth was highly symptomatic, since cutaneous metastases caused a continuous burning sensation that was exacerbated by the contact with liquid and slightly caustic stools at the ileostomy site. Moreover, tumor nodules were oozing and partially bleeding and the abundant exudate engendered continuous troubles in ileostomy management, due to difficult application of the pouching bag. At physical examination, ileostomy outer mucosa appeared macroscopically normal (Figure 2B). Mild stricture was present, but repetitive manual expansions maintained its patency and digital exploration was negative for tumor infiltration. ECT was offered with a palliative intent to improve disease-related complaints and everyday ileostomy management. The patient signed an informed consent. ECT procedure lasted 20 min and was performed under mild general sedation (Figure 3A). Tumor site was rinsed and prepared with sterile drapes; a silicon tube was placed into the ileostomy in order to drain stools and keep the operative field clean. (Figure 3B) Chemotherapy consisted of an intravenous bolus of BLM (15 000 IU/m^2^). After 8 minutes, necessary for drug biodistribution in accordance with the European Standard Operating Procedures on Electrochemotherapy (ESOPE) 15, electric pulses were delivered by percutaneously inserting a 2-cm long needle electrode into skin nodules (Figure 3C). The electrode is composed of seven metal needles arranged in an hexagonal fashion (Figure 1) and is maneuvered by means of an handle connected to an electric pulse generator (Cliniporator^TM^, Igea, Modena, Italy) (Figure 3A). Complete tumor coverage was achieved by means of multiple electrode placements delivering a 1.5 ms -long electric pulse. At the end of the procedure, local toxicity consisted of slight skin marks at the site of electrode insertion (Figure 3D). There was no sign of bleeding, rather the thicker portion of the tumor turned to a bluish coloration due to local vasoconstriction (Figure 3D). After treatment, electroporated skin was carefully cleaned and covered with a healing powder (Figure 4A). Moreover, a custom-sized hydrocolloid dressing (DuoDERM^®^, ConvaTech, Inc.) was applied over the ECT field (Figure 4B) and the space between ileostomy and peristomal skin was sealed by means of a stoma paste (Stomahesive^®^ Paste, ConvaTech, Inc.) in order to prevent stools leakage over inflamed tissues (Figure 3C). Additionally, a silicon catheter was placed into the stoma in order to drain stools directly into the ostomy bag (Figure 4 C,D). The postoperative course was uneventful and the patient was discharged on the same day with the prescription for a course of metronidazole plus ciprofloxacin prophylactic therapy. After one week, tumor nodules were significantly flattened (Figure 5A). Skin care consisted of hydrocolloid dressing application combined with stools deviation by means of a silicon tube (Figure 5B). After three weeks, physical examination showed a normal appearing stoma with adjacent mild erythema and partially necrotic skin from the 12-o’clock to the 7-o’clock position (Figure 5C). Tumor nodules were partially regressed and electroporated skin was healing and dry, thus allowing for easy application and effective sealing of the ostomy flange and pouching bag (Figure 5D). The ileostomy function remained normal throughout this period and no sign of infection was present. In January 2011, one moth after treatment, the patient returned to the outpatient clinic for a minor surgical debridement, during which the necrotic crusts were removed, leaving an underlying intact tissue that was suitable for the application of the ostomy flange. The ileostomy maintained its patency and, importantly, the adherence of the stoma appliance was preserved during the following four months, until patient’s death.

## Discussion

Peristomal tumors represent a rare late complication of surgery for gastrointestinal cancers 16–18 and inflammatory bowel diseases.^19^–^22^ Sporadically, peristomal metastases have been reported also in patients with head and neck 23,24 and genitourinary tumors.^25^ Besides harboring the risk of possible stoma obstruction, these lesions may interfere with peristomal skin care and application of the pouching systems. In patients with bowel deviation, all the conditions that produce a less than perfect adhesion of the stoma appliance may cause direct contact between stools and peristomal skin, thus affecting patient’s quality of life.^26^,^27^ In fact, even minor consequences, such as peristomal dermatitis, need extra medical care that may significantly impair ostomy everyday management, patients’ autonomy and ultimately increase the costs of the procedure.^27^,^29^ Wide local resection followed by stoma resiting, when feasible, represent the only potential effective treatment for peristomal metastases. Unfortunately, in our patient en bloc resection of the stoma together with surrounding soft tissues would have require abdominal wall repair by means of a complex reconstructive technique (*i.e.* long groin flap, anterolateral thigh flap or latissimus dorsi flap) that would have been likely associated with relevant morbidity in a patient with peritoneal carcinomatosis and poor general conditions. On the contrary, ECT procedure proved feasible and well tolerated. It ensured rapid local tumor control thus proving effective in palliating disease-related complaints. A single course of treatment led to an appreciable flattening of peristomal tumor nodules. As a result, the improved peristomal skin conditions allowed for the effective application of ileostomy bag and prevented stools spillage on the abdominal wall.

Since local inflammatory reaction and soft tissue ulceration are possible side effects of ECT application 30,31, one challenging aspect was played by peristomal skin protection from the ileostomy outflow. Our management strategy aimed at separating the electroporated skin from stools. For this purpose, a smooth silicon drain was placed into the ileostomy during the procedure and the first postoperative course. Moreover, a hydrocolloid dressing proved to be an effective barrier and a reliable support for the flange of the pouching system.

It is not clear to us why, despite the rapid and homogeneous tumor response after one week (Figure 4A), afterwards local response was rather inhomogeneous and some portions of the tumor were clearly necrotic, while others not (Figure 4C). Although BLM was given intravenously, in accordance with ESOPE guidelines 14 which recommend systemic drug administration in case of multiple or large tumors, the irregular tumor vascularization and consequent inhomogeneous drug distribution could explain the jeopardized response to treatment in our patient.

ECT has been recognized as a safe and effective option for patients with superficially disseminated metastases, mainly from malignant melanoma and breast cancer.^30^,^31^ Interestingly, several other tumor histotypes seem to be sensitive to electroporation-driven chemotherapy, thus potentially expanding the number of eligible patients.^11^ If its efficacy and feasibility will be further confirmed, ECT could represent not only a valuable palliative option for peristomal metastases, but also an effective treatment for those patients with primary skin / soft tissue tumors arising at any ostomy site. Although rare, the occurrence of peristomal primary tumors have been described, especially in the setting of colorectal cancer or inflammatory bowel diseases (Table 1).^32^–^38^

Waiting for more evidence, in our opinion some precautions should be taken when applying ECT to peristomal lesions. First, since some peristomal tumors may represent metachronous intestinal cancers, which are not amenable with current ECT equipment (due to feasibility of electrode application and possible damage to intestinal wall), pre-operative clinical or endoscopic evaluation seems mandatory. Second, electrode insertion should be extremely careful and possibly image-guided, in order to avoid direct physical damage or late cytotoxic effects to the stoma. Finally, the assistance of an ostomy nursing staff represent a precious support to ensure adequate skin care and pouching system management.

In conclusion, we applied, for the first time, ECT in a very rare, but morbid condition such as skin tumor infiltration from GC at an ileostomy site. A single 20-minute treatment course led to rapid tumor control, prevented stoma obstruction and improved peristomal skin conditions, thus ensuring effective application of the stoma flange and its long-term (as in a palliative setting) maintenance, without stools leakage. These results significantly improved patient’s quality of life.

## Figures and Tables

**TABLE 1.** Primary peristomal skin/soft tissues tumors: summary of case reports from literature review

**Author (year)**	**Disease**	**Ostomy**	**Interval (years)**	**Clinical presentation**	**Histotype**	**Treatment**	**Follow-up**
Didolkar 33 (1975)	RC	C	33	4-cm size peristomal ulceration	BCC	WLE + stoma resiting + split-thickness skin graft	2.5 years
O’Connell 34 (1987)	CD	I	39	painful peristomal ulceration, 3.0 × 2.5 fungating lesion	SCC	WLE + stoma resiting + skin flap transposition	3 months
Wu 35 (2000)	UC	I	44	Painful, 10-cm size, peristomal skin lesion	SCC	WLE + stoma resiting	1 year
Ramanujam 36 (2002)	UC	I	51	4×3 cm peristomal ulcer, peristomal discomfort and bleeding	SCC	WLE + stoma resiting	28 months
Sewell 37 (2008)	RC	C	33	slowly growing, ulcerated parastomal nodule	BCC	MMS	16 years
Fessa 38 (2010)	RC	C	12	progressive erythematous patch	AS	WLE + stoma resiting	nr
Liu ^39^ (2012)	UC	I	33	oozing peristomal erosions, difficult adherence of stoma appliance	Paget	WLE + stoma revision	nr

Abbreviations: RC = rectal carcinoma; CD = Crohn disease; C = colostomy; BCC = basal cell carcinoma; WLE = wide local excision; I = ileostomy; SCC = squamous cell carcinoma, UC = ulcerative colitis; MMS = Mohs micrographic surgery, AS = angiosarcoma; nr = not reported

## References

[1] Guggenheim DE, Shah MA (2013). Gastric cancer epidemiology and risk factors. J Surg Oncol.

[2] Gates O (1937). Cutaneous metastases of malignant disease. Am J Cancer.

[3] Alcaraz I, Cerroni L, Rütten A, Kutzner H, Requena L (2012). Cutaneous metastases from internal malignancies: a clinicopathologic and immunohistochemical review. Am J Dermatopathol.

[4] Takata T, Takahashi A, Tarutani M, Sano S (2013). A rare case of cellulitis-like cutaneous metastasis of gastric adenocarcinoma. Int J Dermatol.

[5] Qiao J, Fang H (2012). Cutaneous nodule in a young man. JAMA.

[6] Ozakyol AH, Sariçam T, Paşaoğlu O (1999). A rare entity: cutaneous metastasis from gastric adenocarcinoma. Am J Gastroenterol.

[7] Sood A, Midha V, Sekhon JS, Sidhu SS (1998). Generalized cutaneous metastases from carcinoma stomach. Am J Gastroenterol.

[8] Choi HM, Myung KB, Kook HI (1986). Cutaneous metastatic adenocarcinoma of stomach: nodular and inflammatory carcinoma. J Korean Med Sci.

[9] Früh M, Ruhstaller T, Neuweiler J, Cerny T (2005). Resection of skin metastases from gastric carcinoma with long-term follow-up: an unusual clinical presentation. Onkologie.

[10] García-González E, Alvarez-Paque L, Loyola-Zárate M, Lisker-Melman M (1998). Seeding of gastric adenocarcinoma cells to the skin after invasive procedures. J Clin Gastroenterol.

[11] Mali B, Jarm T, Snoj M, Sersa G, Miklavcic D (2013). Antitumor effectiveness of electrochemotherapy: a systematic review and meta-analysis. Eur J Surg Oncol.

[12] Sadadcharam M, Soden DM, O’sullivan GC (2008). Electrochemotherapy: an emerging cancer treatment. Int J Hyperthermia.

[13] Gothelf A, Mir LM, Gehl J (2003). Electrochemotherapy: results of cancer treatment using enhanced delivery of bleomycin by electroporation. Cancer Treat Rev.

[14] Mir LM, Gehl J, Sersa G, Collins CG, Garbay JR, Billard V (2006). Standard operating procedures of the Electrochemotherapy: instructions for the use of bleomycin or cisplatin administered either systemically or locally and electric pulses delivered by the Cliniporator by means of invasive or noninvasive electrodes. EJC Suppl.

[15] Cava A, Román J, González Quintela A, Martín F, Aramburo P (1990). Subcutaneous metastasis following laparoscopy in gastric adenocarcinoma. Eur J Surg Oncol.

[16] Townley WA, Kothari MS, Meyrick-Thomas J (2005). Metachronous stomal adenocarcinoma following abdominoperineal resection for rectal cancer. Ann R Coll Surg Engl.

[17] Greenberg HL, Lopez L, Butler DF (2006). Peristomal metastatic adenocarcinoma of the rectum. Arch Dermatol.

[18] Vijayasekar C, Noormohamed S, Cheetham MJ (2008). Late recurrence of large peri-stomal metastasis following abdomino-perineal resection of rectal cancer. World J Surg Oncol.

[19] Cuesta MA, Donner R (1976). Adenocarcinoma arising at an ileostomy site: report of a case. Cancer.

[20] Smart PJ, Sastry S, Wells S (1988). Primary mucinous adenocarcinoma developing in an ileostomy stoma. Gut.

[21] Quah HM, Samad A, Maw A (2005). Ileostomy carcinomas a review: the latent risk after colectomy for ulcerative colitis and familial adenomatous polyposis. Colorectal Dis.

[22] Achneck HE, Wong IY, Kim PJ, Fernandes MA, Walther Z, Seymour NE, Jain D (2005). Ileostomy adenocarcinomas in the setting of ulcerative colitis. J Clin Gastroenterol.

[23] el Shennawy M, Fayek A, el Sharkawy L (2000). A study of peristomal recurrence. Rev *Laryngol Otol Rhinol (Bord)*.

[24] Fagan JJ, Loock JW (1996). Tracheostomy and peristomal recurrence. Clin Otolaryngol Allied Sci.

[25] Yoshida T, Takayama H, Uemura M, Nakai Y, Nonomura N, Tsujimura A (2012). Solitary skin metastasis adjacent to ureterocutaneostomy 4 years after radical cystectomy for bladder cancer. Jpn J Clin Oncol.

[26] Scarpa M, Ruffolo C, Boetto R, Pozza A, Sadocchi L, Angriman I (2010). Diverting loop ileostomy after restorative proctocolectomy: predictors of poor outcome and poor quality of life. Colorectal Disease.

[27] Scarpa M, Sadocchi L, Ruffolo C, Iacobone M, Filosa T, Prando D (2007). Rod in loop ileostomy: just an insignificant detail for ileostomy-related complications?. Langenbecks Arch Surg.

[28] Carlsen E, Bergen AB (1999). Loop ileostomy: technical aspects and complications. Eur J Surg.

[29] McLeod RS, Lavery IC, Leatherman JR, Maryland PA, Fazio VW, Jagelman DG (1986). Factors affecting quality of life with a conventional ileostomy. World J Surg.

[30] Campana LG, Mocellin S, Basso M, Puccetti O, De Salvo GL, Chiarion-Sileni V (2009). Bleomycin-based electrochemotherapy: clinical outcome from a single institution’s experience with 52 patients. Ann Surg Oncol.

[31] Campana LG, Valpione S, Falci C, Mocellin S, Basso M, Corti L (2012). The activity and safety of electrochemotherapy in persistent chest wall recurrence from breast cancer after mastectomy: a phase-II study. Breast Cancer Res Treat.

[32] Didolkar MS, Douglass HO, Holyoke ED, Elias EG (1975). Basal-cell carcinoma originating at the colostomy site: report of a case. Dis Colon Rectum.

[33] O’Connell PR, Dozois RR, Irons GB, Scheithauer BW (1987). Squamous cell carcinoma occurring in a skin-grafted ileostomy stoma. Report of a case. Dis Colon Rectum.

[34] Wu JS, Sebek BA, Fazio VW (2000). Images for surgeons. Parastomal squamous cell carcinoma in an ileostomy 44 years after proctocolectomy. J Am Coll Surg.

[35] Ramanujam P, Venkatesh KS (2002). An unusual case of squamous cell carcinoma arising at the stomal site: case report and review of the literature. J Gastrointest Surg.

[36] Sewell LD, Marks VJ (2008). Excision of a peristomal basal cell carcinoma using Mohs micrographic surgery. Dermatol Surg.

[37] Fessa CK, Sharma R, Fernández-Peñas P (2010). Cutaneous epithelioid angiosarcoma occurring at a peristomal site. J Am Acad Dermatol.

[38] Liu X, Melton GB, Xie H, Dietz DW (2012). Extramammary Paget disease in peristomal skin: report of a unique case. J Gastrointest Surg.

